# Identification and Quantification of Polyphenols in Croatian Traditional Apple Varieties

**DOI:** 10.3390/plants11243540

**Published:** 2022-12-15

**Authors:** Ana-Marija Gotal Skoko, Bojan Šarkanj, Marta Lores, Maria Celeiro, Martina Skendrović Babojelić, Dragutin Kamenjak, Ivana Flanjak, Antun Jozinović, Tihomir Kovač, Ante Lončarić

**Affiliations:** 1 Faculty of Food Technology Osijek, Josip Juraj Strossmayer University of Osijek, Franje Kuhača 18, 31000 Osijek, Croatia; 2 Department of Food Technology, University Centre Koprivnica, University North, Trg dr. Žarka Dolinara 1, 48000 Koprivnica, Croatia; 3 LIDSA-CRETUS Department of Analytical Chemistry, University of Santiago de Compostela, E-15782 Santiago de Compostela, Spain; 4 Department of Pomology, Faculty of Agriculture, University of Zagreb, Svetošimunska 25, 10000 Zagreb, Croatia; 5 Križevci College of Agriculture, M. Demerca 1, 48260 Križevci, Croatia

**Keywords:** Croatian traditional apple varieties, LC-MS/MS, polyphenols, bioactive compounds

## Abstract

Apples and apple-based products are particularly interesting due to being a good source of polyphenols in an everyday diet. Recently there has been increased interest in the preservation of traditional apple varieties due to studies that suggest that traditional apple varieties have a higher content of polyphenols and antioxidant activity compared to commercial varieties. This study shows that traditional apple varieties contain higher concentration of polyphenolic compounds than conventional ones, such as chlorogenic acid (1.29–456 mg/kg dw), catechin (0.70–312 mg/kg dw), epicatechin (1.75–244 mg/kg dw), procyanidin B1 + B2 (4.08–358 mg/kg dw) and quercetin-3-glucoside (0.96–231 mg/kg dw). This research underlies the great potential of traditional apple varieties as a source of natural antioxidants and polyphenolics.

## 1. Introduction

Apples (family Rosaceae, species *Malus domestica* Borkh) and apple-based products are one of the most consumed fruits around the world and are significant sources of varied biologically active compounds, such as polyphenols [1]. Lately, there has been a growing interest in the preservation of traditional apple varieties, which are locally adapted to their natural environments, including specific regions, small orchards and homegrown [2]. This interest has grown due to studies that propose that traditional apple varieties contain higher levels of polyphenols and antioxidant activity compared to commercial ones [3]. Furthermore, fresh fruit consumption represents the main source of polyphenols, and apples are generally available throughout the year [4]. Polyphenols are naturally occurring secondary metabolite phytochemicals produced in plant sources [5]. Moreover, they can be found bound in the cell wall and they represent more than 50% of non-extractable polyphenols in some plants [6]. Apples contain several main groups of polyphenolic compounds: flavanols, phenolic acids, dihydrochalcones, flavonols and anthocyanins [7]. Traditional apple varieties prove to be rich in some individual polyphenols, such as quercetin-3-rutinoside, procyanidins B1, B2, A2, epicatechin, chlorogenic acid, etc. [8]. The by-products of apple processing (pomace and peel) might also be used as a source of polyphenols [9]. Wang et al. [10] reported that the content of polyphenols in apple pomace (120–300 mg/100 g dw) were much higher than those in apple tissue, apple flesh and apple juice. Bars-Cortina et al. [11] reported that epicatechin and its polymerized forms such as dimer, trimer and tetramer were the main flavan-3-ols detected in the apple peel, with a high variability between apple varieties. The content of apple polyphenols is also influenced by water conditions of the harvesting season, fruit maturity, cultivar properties, geographic location, etc. Moreover, it was found that anthocyanins in the apple peel can be increased by environmental factors, such as temperature and light [12]. On the other hand, procyanidins and flavan-3-ols are reduced in the maturation process, which is similar to other polyphenols [13]. For example, red-fleshed apples contained higher amounts of chlorogenic acid (238 ± 35.7 mg/kg), anthocyanins (49.2 ± 6.55 mg/kg), dihydrochalcones (47.8 ± 4.67 mg/kg) and organic acid, but lower amount of flavan-3-ols (21.9 ± 0.96 mg/kg) than white-fleshed apples [11]. The concentration of procyanidins is significantly correlated with antioxidant activity and various clinical studies have suggested that procyanidins and flavan-3-ols are entangled in various health benefits, such as prevention of cardiovascular diseases, type II diabetes, dementia and cancer [13,14,15]. Furthermore, their antioxidant potential depends on the chain length of oligomers and the type of oxygen they react with. This also applies to procyanidins and their monomer [16,17]. Starowicz et al. [18] reported that procyanidins are stronger antioxidants than more absorbable epicatechin and catechin. In the present study, LC-MS/MS analysis was employed to determine polyphenols in the analysed samples, allowing the quantification of catechin, procyanidin B1, procyanidin B2, chlorogenic acid, epicatechin, quercetin-3-rutinoside, quercetin-3-glucoside and astragalin in a large number of traditional apple varieties. Despite the nutritional value, some polyphenols possess antifungal, antioxidant activity, anti-inflammatory, antiviral, antitoxin, anticancer properties, etc. For example, catechin has antioxidant, antibacterial and antiviral activity [19]. Moreover, catechin and epicatechin were isolated as antifungal compounds [20]. Astragalin has antidiabetic, anticancer, anti-inflammatory and antioxidant activity [21] Quercetin-3-rutinoside is an anti-allergic, antimicrobial and antifungal agent [22,23]. Furthermore, chlorogenic acid is known as an antioxidant and radical scavenger. It also has antifungal properties that were tested in vitro against pathogenic fungi [24]. Additionally, in this work, the total polyphenolic content (TPC) and antioxidant activity using DPPH radical were assessed. The purpose of this research was the identification and quantification of polyphenols in examined apples; to determine the total polyphenolic content and antioxidant activity; and to determine the content of astragalin in traditional apple varieties, which, to the best of our knowledge, is the first study to do so.

## 2. Results and Discussion

In this study, the quantification and identification of polyphenols using LC-MS/MS, total polyphenolic content (TPC) and antioxidant activity in selected Croatian traditional apple varieties was performed. The TPC values in traditional apple varieties are given in Table 1. The highest TPC was detected in ‘Bobovec’ (13,264.9 ± 10.2 mg/kg dw), followed by ‘Ilinjača’ (10,806.6 ± 68.3 mg/kg dw) and ‘Ploska Letovanička’ (10,126.7 ± 2.7 mg/kg dw). The lowest TPC was found in ‘Kraljevača’ (3781.5 ± 14.6 mg/kg dw). These results are in accordance with those reported by Akagić et al. [25] and Skoko et al. [12], where the highest TPC in traditional apple varieties was observed for ‘Srčika’ (813.9 ± 11.8 mg/kg dw), followed by ‘Adamovka’ (613.0 ± 4.0 mg/kg dw) varieties. On the other hand, in the same research, commercial apple varieties showed concentrations of total polyphenols in the range from 437.0 to 286.4 mg/kg dw. In conclusion, traditional apple varieties contain a higher content of total polyphenols than commercial ones.

**Figure 1:**
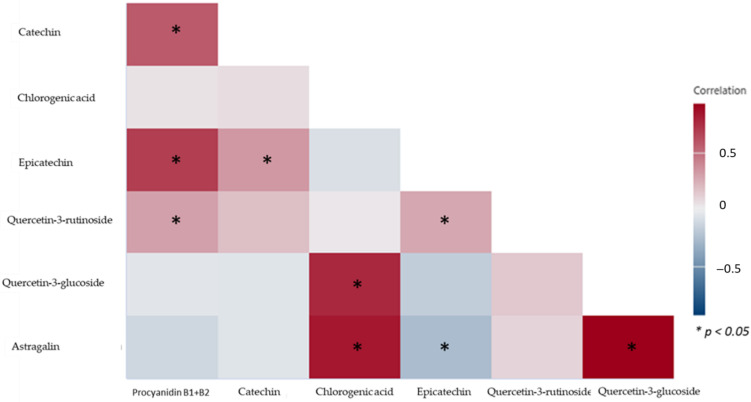
The colour map of correlations between polyphenols in 23 Croatian traditional apple varieties, * statistically significant negative or positive correlation.

**Figure A1:**
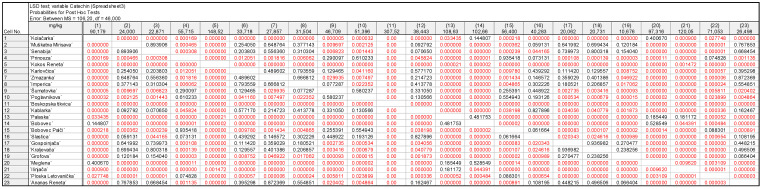
The statistical significance of catechin in traditional apple varieties; the *p*-values of the correlations of identified polyphenols. Statistically significant *p*-values are marked red (*p* < 0.05).

**Figure A2:**
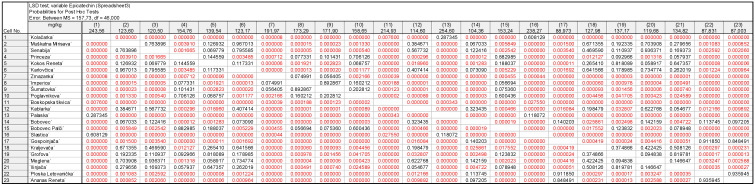
The statistical significance of epicatechin in traditional apple varieties; the *p*-values of the correlations of identified polyphenols. Statistically significant *p*-values are marked red (*p* < 0.05).

**Figure A3:**
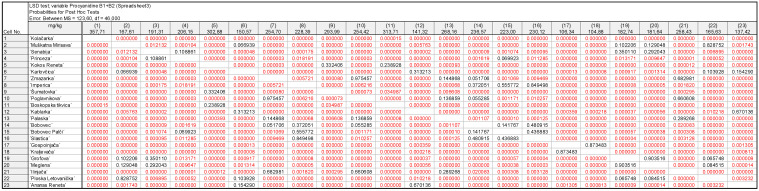
The statistical significance of procyanidin B1 + B2 in traditional apple varieties; the *p*-values of the correlations of identified polyphenols. Statistically significant *p*-values are marked red (*p* < 0.05).

**Figure A4:**
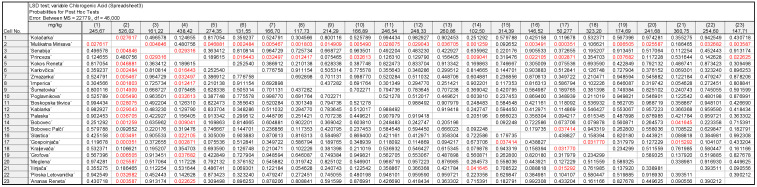
The statistical significance of chlorogenic acid in traditional apple varieties; the *p*-values of the correlations of identified polyphenols. Statistically significant *p*-values are marked red (*p* < 0.05).

**Figure A5:**
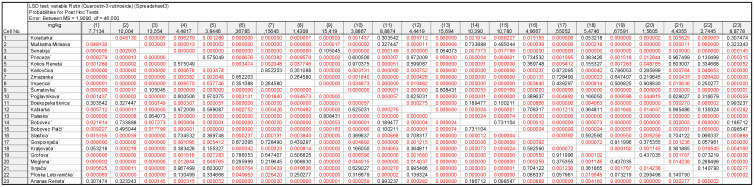
The statistical significance of quercetin-3-rutinoside in traditional apple varieties; the *p*-values of the correlations of identified polyphenols. Statistically significant *p*-values are marked red (*p* < 0.05).

**Figure A6:**
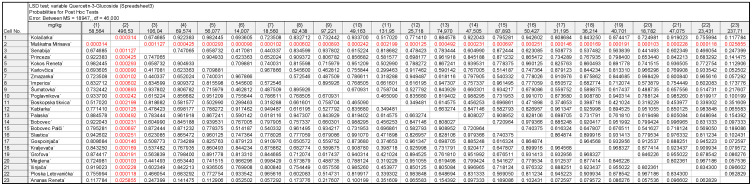
The statistical significance of quercetin-3-glucoside in traditional apple varieties; the *p*-values of the correlations of identified polyphenols. Statistically significant *p*-values are marked red (*p* < 0.05).

**Figure A7:**
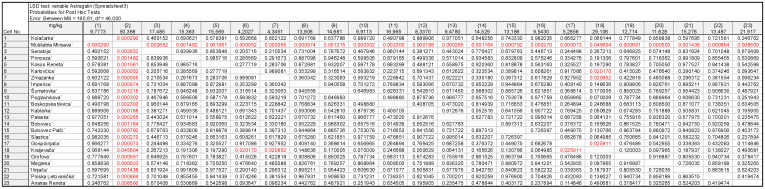
The statistical significance of astragalin in traditional apple varieties; the *p*-values of the correlations of identified polyphenols. Statistically significant *p*-values are marked red (*p* < 0.05).

Furthermore, the content of the most abundant polyphenols found in apples, catechin and epicatechin, were determined (Table 2). The highest content of catechin was detected in ‘Boskopska tikvica’ (311.79 ± 18.1 mg/kg dw), followed by a significantly lower content of catechin in ‘Kokos Reneta’ (145.19 ± 8.5 mg/kg dw). The highest content of epicatechin was detected in ‘Kolačarka’ (243.56 ± 4.4 mg/kg dw), followed by a similar content in ‘Slastica’ (243.16 ± 28.5 mg/kg dw). On the other hand, the lowest content of catechin and epicatechin was detected in ‘Gospoinjača’ (0.70 ± 0.2 mg/kg dw: 1.75 ± 0.0 mg/kg dw, respectively). The statistical significance of catechin and epicatechin is shown in Figure A1 and Figure A2. These results are comparable to the content of catechin and epicatechin reported in our previous study [26], where the content of epicatechin in all traditional apple varieties was higher than the content of catechin in the same sample. The same trend was observed in all of the examined samples in this study. For example, the content of epicatechin for ‘Palaska’ was 231.27 ± 41.8 mg/kg dw, whereas the content of catechin was significantly lower (95.29 ± 14.7 mg/kg dw). Procyanidin B1 and B2 were also detected in all of the examined traditional apple varieties (Table 2). Procyanidins are the major class of apple polyphenols, and they are stronger antioxidants than epicatechin and catechin. The highest content of procyanidin B1 + B2 was detected in ‘Kolačarka’ (357.71 ± 35.2 mg/kg dw), followed by approximately similar content in ‘Boskopska tikvica’ (313.03 ± 1.5 mg/kg dw). Such statistical significance is shown in Figure A3. Chlorogenic acid, the polyphenol that is also responsible for higher antioxidant activity in apples, was also detected in the apple samples. The highest content of chlorogenic acid was detected in ‘Princeza’ (455.86 ± 2.1 mg/kg dw), followed by ‘Ilinjača’ (388.38 ± 22.7 mg/kg dw). On the other hand, the lowest content of procyanidin B1 + B2 and chlorogenic acid was detected in ‘Gospoinjača’ (4.08 ± 0.2 mg/kg dw; 1.29 ± 0.2 mg/kg dw, respectively). The statistical significance of chlorogenic acid is shown in Figure A4. These results of procyanidin B1 + B2 and chlorogenic acid are in accordance with those previously reported by Raudone et al., [27]. Furthermore, quercetin-3-rutinoside and quercetin-3-glucoside were also detected in all of the apple samples. The content of quercetin-3-rutinoside and quercetin-3-glucoside was also measured in traditional apple varieties. The results of flavonols are presented in Table 2. The highest content of quercetin-3-rutinoside was measured in ‘Palaska’ (15.69 ± 1.6 mg/kg dw), followed by ‘Šumatovka’ (14.75 ± 1.6 mg/kg dw). The lowest content was measured in ‘Gospoinjača’ (0.15 ± 0.0 mg/kg dw). The highest content of querce-tin-3-glucoside was measured in ‘Ananas Reneta’ (230.66 ± 15.8 mg/kg dw), followed by significantly lower content in ‘Boskopska tikvica’ (132.19 ± 8.2 mg/kg dw). On the other hand, the lowest content of quercetin-3-glucoside was measured in ‘Gospoinjača’ (0.96 ± 0.1 mg/kg dw). The obtained results show that all traditional apple varieties have a significantly higher content of quercetin-3-glucoside than the content of quercetin-3-rutinoside. The statistical significance of quercetin-3-rutinoside and quercetin-3-glucoside is shown in Figure A5 and Figure A6. Other authors also observed that the two groups of apple varieties could be defined. The first group can be defined by a higher content of chlorogenic acid and flavan-3-ols and the second group can be defined by great amounts of quercetin derivatives [28,29]. Moreover, quercetin-3-rutinoside has been used conventionally as an antiallergic, antimicrobial and antifungal agent, and certain research has shown pharmacological benefits for the treatment of different chronic diseases such as hypertension, cancer and diabetes [23]. The content of astragalin, a naturally occurring flavonoid, has also been detected in all traditional apple varieties (Table 2). The richest source of astragalin with 29–34% of total polyphenols as compared to other plants was *Cercis chinensis* [30]. Furthermore, astragalin possesses a broad spectrum of pharmacological properties, such as neuroprotective, antidiabetic, antioxidant, anticancer and anti-inflammatory [31,32]. The obtained results show that the highest content of astragalin was measured in ‘Kraljevača’ (28.79 ± 0.3 mg/kg dw), followed by ‘Ananas Reneta’ (21.79 ± 1.1 mg/kg dw). The statistical significance of astragalin is shown in Figure A7. It should be highlighted that ‘Gospoinjača’ had the lowest content of all examined polyphenols, such as astragalin (0.70 ± 0.1 mg/kg dw), quercetin-3-rutinoside, quercetin-3-glucoside, procyanidin B1 + B2, chlorogenic acid, epicatechin and catechin. Research about the presence of astragalin, as far as we know, was identified for the first time in traditional apple varieties. On the other hand, astragalin could undergo structural optimization to improve its chemical availability and optimize its absorption profiles, which will ultimately lead to potential drug candidates [21]. Furthermore, the colour map of correlations between all polyphenols in 23 Croatian traditional apple varieties could be found in Figure 1.

The antioxidant activity (AA) of traditional apple varieties was determined using DPPH reagent, which is one of the most used in vitro assays, based on the inactivation of stable synthetic radicals. Table 3 presents the results of the AA of twenty-three traditional apple varieties. The highest antioxidant activity was measured in ‘Meglena’ (59.0 ± 3.6 mmol TE/kg dw), followed by ‘Ploska Letovanička’ (58.6 ± 3.4 mmol TE/kg dw). It should be highlighted that ‘Ploska Letovanička’ also was one of the varieties with the highest TPC, which is mainly responsible for the higher AA. On the other hand, the lowest AA was measured in ‘Muškatna Mirisava’ (11.3 ± 0.4 mmol TE/kg dw). These results are comparable to the AA reported in our previous study [26]. 

The highest content of procyanidin B1 + B2 was negatively correlated with the higher content of quercetin-3-glucoside and astragalin. This means that increasing the content of quercetin and astragalin leads to a decrease in the content of procyanidin B1 + B2. This is in accordance with the previously stated facts that traditional apples varieties have a higher content of procyanidin B1 + B2, and therefore a lower content of astragalin and quercetin-3-glucoside. Furthermore, as far as we know, astragalin, which has a significant antifungal effect, was identified for the first time in traditional apple varieties and it is positively correlated with phenolic acids; therefore, apples with a higher content of phenolic acids will also contain a higher content of astragalin. The higher content of procyanidin B1 + B2 was positively correlated with the higher content of catechin, epicatechin and quercetin-3-rutinoside (*p* < 0.05). The highest content of catechin was negatively correlated with a higher content of astragalin and quercetin-3-glucoside, and positively correlated with a higher content of epicatechin (*p* < 0.05). The highest content of chlorogenic acid was negatively correlated with a higher content of epicatechin and positively correlated with a higher content of quercetin-3-glucoside and astragalin (*p* < 0.05). On the other hand, the highest content of epicatechin was negatively correlated with a higher content of quercetin-3-glucoside and astragalin, and positively correlated with a higher content of quercetin-3-rutinoside. The highest content of quercetin-3-rutinoside was positively correlated with a higher content of quercetin-3-glucoside and astragalin. The highest content of quercetin-3-glucoside was positively correlated with a higher content of astragalin (*p* < 0.05). Such correlation is important because it helps us understand the difference between individual polyphenols, polyphenols, mycotoxins, etc. For example, the apple varieties with a higher content of catechin and epicatechin induce patulin production [26].

## 3. Material and Methods

### 3.1. Chemicals and Reagents

The determined polyphenols, their CAS numbers, retention time and MS/MS transitions are summarized in Table 4. Methanol (MeOH) and ultrapure water, both MS grade, were supplied by Scharlab (Barcelona, Spain). Folin–Ciocalteu’s phenol reagent (2M), 2,2-diphenyil-1-picrylhydrazyl (DPPH), 6-hydroxy-2,5,7,8-tetramethylchroman-2-carboxylic acid (Trolox^®^) and formic acid were purchased from Sigma–Aldrich (Darmstadt, Germany). Sodium carbonate (Na_2_CO_3_) was supplied by Panreac (Barcelona, Spain). All chemicals and reagents were of analytical grade.

The individual standard stock solutions for each polyphenol (500–1000 µg/mL) were prepared in methanol. Further dilutions and mixtures were prepared in MeOH/water (50:50, *v*/*v*). The calibration curves were prepared in MeOH/water (50:50, *v*/*v*) covering a concentration range between 0.1 and 5 µg/ mL for all target compounds, excluding procyanidins (1–20 µg/mL). All solutions were stored at −20 °C and protected from light.

### 3.2. Plant Material

The fruits of traditional apple varieties: ‘Kolačarka’, ‘Muškatna Mirisava’, ‘Senabija’, ‘Princeza’, ‘Kokos Reneta’, ‘Karlovčica’, ‘Zmazanka’, ‘Imperica’, ‘Šumatovka’, ‘Poglavnikova’, ‘Boskopska tikvica’, ‘Kablarka’, ‘Palaska’, ‘Bobovec’, ‘Bobovec Palči’, ‘Slastica’, ‘Gospoinjača’, ‘Kraljevača’, ‘Grofova’, ‘Meglena’, ‘Ilinjača’, ‘Ploska Letovanička’ and ‘Ananas Reneta’ were collected in October 2021 at the College of Agriculture in Križevci (46°01′36.1″ N 16°33′16.3″ E), Croatia. The orchard was planted in 2014 by grafting traditional apple varieties to the rootstock MM 106 (the origin of the buds of traditional varieties was from the collection plantation of the family farm Horvatić, Cvetkovec, Croatia 46°20′85.9″ N 16°69′86.4″ E). The planting distance between the rows is 4 m and within each row is 2 m. Each variety is represented by three trees. The rows are reinforced with columns and wires, and the cultivation form is an irregular palmette. The soil type is pseudogley, slightly acidic to neutral reacting, loamy-clay texture, slightly humic and very well supplied with phosphorus and potassium, and the orchard is located in an area with temperate continental climate. All twenty-three apple varieties studied were authenticated by a pomologist. The average sample was prepared from 5 apples of each variety, which were previously lyophilized (Christ, Osterode am Harz, Germany) and pulverized. A total of 250 mg of the average sample was mixed with 1,25 mL of extraction solvent (80% aqueous methanol). Furthermore, extraction of bioactive polyphenolic compounds was performed by Ultrasound extraction (UAE) in an ultrasonic bath at 35 kHz for 15 min in 20 mL test tubes. After the extraction, the obtained extracts were centrifuged (Heareus, Multifuge 3 L-R Centrifuge, Thermo Fisher Scientific, San Jose, CA, US) at 25 °C for 15 min and filtered through a 0.45 µm polytetrafluoroethylene (PTFE) syringe-tip filter (Chromanfil Xtra, Macherey-Nagel GmbH & Co. KG, Düren, Germany). Each extraction was performed in triplicate.

### 3.3. Total Polyphenolic Content (TPC) Determination

The total polyphenolic content (TPC) was determined employing the Folin–Ciocalteu (FC) colorimetric method described by Singleton and Rossi [33], employing a modification of the Zhang’s guidelines [34] for microtitration in 96-well plates. Briefly, 20 µL of the diluted extract was mixed with 100 µL of Folin–Ciocalteu reagent (1:10, *v*/*v*) and 80 µL of sodium carbonate solution (7.5 g/L). The mixture was shaken and kept in the dark for 30 min. Afterwards, the absorbance was measured at 760 nm in a microplate reader (BMG LABTECH, Ortenberg, Germany). The TPC was quantified employing a calibration curve of gallic acid covering a concentration range between 20 and 160 mg/L. The TPC was expressed as milligrams of gallic acid equivalent (mg GAE) per kg of dry weight of apple.

### 3.4. Antioxidant Activity (AA) Determination

The antioxidant activity (AA) was determined using the DPPH reagent following the method described by Symes et al., [35]. Briefly, 100 µL of the extracts at different dilutions factors were placed in a 96-well plate and mixed with 100 µL of DPPH reagent (prepared in methanol). The mixture was kept in the dark for 10 min and the measurement was performed at 515 nm in a microplate reader (BMG LABTECH, Ortenberg, Germany). For the quantification of the AA, a calibration curve prepared in Trolox, covering a concentration range between 3 and 31 mg/kg, was employed. The AA was expressed as millimoles Trolox equivalent (mmol TE) per kg of dry weight of apple.

### 3.5. LC-MS/MS Analysis

For the identification and quantification of the target polyphenols in the apple samples, LC-MS/MS analysis was performed employing a Thermo Scientific (San José, CA, USA) instrument based on a TSQ Quantum UltraTM triple quadrupole mass spectrometer equipped with a HESI-II (heated electrospray ionization) source and an Accela Open autosampler with a 10 μL loop. The chromatographic separation was performed using a Kinetex C18 column (2.6 μm, 100 × 2.1 mm) with a guard column (SecurityGuardTM ULTRA Holder) obtained from Phenomenex (Torrance, CA, USA). The column temperature was set at 50 °C and the injection volume was 10 μL. The mobile phase consisted of water (A) and methanol (B), both containing 0.1 % formic acid. The eluted program started with 5% of B (held 5 min), and increased to 90% of B over 11 min (held 3 min). Then, initial conditions were reached in 5 min. The flow rate was kept constant at 200 μL/min. The total run time for each injection was 20 min. The mass spectrometer and the HESI-II source were working simultaneously in the positive and negative mode. Selected Reaction Monitoring (SRM) acquisition mode was implemented monitoring 2 or 3 transitions per compound (Table 1) for an unequivocal identification and quantification of the target compounds. The system was operated by Xcalibur 2.2 and Trace Finder^TM^ 3.2.

### 3.6. Statistical Analysis

The data presented in this work are expressed as the mean value ± SEM (standard error of measurement) from three separate experiments. The pooled datasets were checked for distribution normality by the Shapiro–Wilk test and homoscedasticity was checked by the Levene test and compared by analysis of variance (ANOVA). The ANOVA test showed that there were statistically significant differences, therefore we employed the LSD test to determine the exact differences. Statistical analyses were performed using Statistica 13.5 (TIBCO Software Inc., Palo Alto, CA, USA) and the differences were considered statistically significant when the *p* value was <0.05. A correlogram was prepared by using Minitab Statistical Software (Minitab LLC, State College, PA, USA), while statistically significant correlations were checked by Statistica 13.5.

## 4. Conclusions

The research conducted within this study provides comprehensive data on the polyphenol profiles of Croatian traditional apple varieties. It was demonstrated that traditional apple varieties have higher content of polyphenolic compounds and antioxidant activity than classical ones. In this study, several polyphenols with interesting properties, such as chlorogenic acid, catechin, epicatechin, procyanidin B1 + B2 and quercetin-3-glucoside were quantified, highlighting the presence of quercetin-3-rutinoside at high concentrations, up to 16 mg/kg dw. Additionally, astragalin, which has a significant antifungal effect, was quantified for the first time in traditional apples, reaching concentrations up to 29 mg/kg dw in the ‘Kraljevača’ variety. Furthermore, quercetin-3-rutinoside has been used conventionally as an antimicrobial, antifungal and antiallergic agent, and some research has shown it has pharmacological benefits in the treatment of various chronic diseases. Future research will be performed to contribute to food safety from the point of view of contributing to global food sufficiency and help preventing economic losses.

## Figures and Tables

**Table 1:** Total polyphenol content (mg/kg dw) in traditional apple varieties.

Sample ID	mg/kg dw
‘Kolačarka’	8891 ± 144.1
‘Muškatna Mirisava’	4062.8 ± 29.1
‘Senabija’	4827.3 ± 0.9
‘Princeza’	7603.6 ± 109.9
‘Kokos Reneta’	5416.1 ± 136.5
‘Karlovčica’	4414.9 ± 210.5
‘Zmazanka’	4494.1 ± 61.8
‘Imperica’	3823.1 ± 4.0
‘Šumatovka’	8468.9 ± 321.0
‘Poglavnikova’	5518.3 ± 46.2
‘Boskopska tikvica’	6743.5 ± 99.2
‘Kablarka’	5788.1 ± 2.6
‘Palaska’	9660.4 ± 220.0
‘Bobovec’	13264.9 ± 10.2
‘Bobovec Palči’	6424.7 ± 168.9
‘Slastica’	5230.5 ± 116.5
‘Gospoinjača’	4951.6 ± 2.8
‘Kraljevača’	3781.5 ± 14.6
‘Grofova’	4317.9 ± 130.1
‘Meglena’	8995.9 ± 26.0
‘Ilinjača’	10,806.6 ± 68.3
‘Ploska Letovanička’	10,126.7 ± 2.7
‘Ananas Reneta’	6869.3 ± 79.8

Mean ± SD based on two extracts each measured twice (*n* = 4).

**Table 2:** Content of polyphenols (mg/kg dw ^a^) in traditional apple varieties.

Sample ID	Catechin (mg/kg dw)	Epicatechin (mg/kg dw)	Procyanidin B1 + B2 (mg/kg dw)	Chlorogenic Acid (mg/kg dw)	Quercetin-3-Rutinoside (mg/kg dw)	Quercetin-3-Glucoside (mg/kg dw)	Astragalin (mg/kg dw)
‘Kolačarka’	90.18 ± 7.5	243.56 ± 4.4	357.71 ± 35.2	245.67 ± 40.6	7.71 ± 0.7	58.56 ± 5.0	9.78 ± 1.8
‘Muškatna Mirisava’	30.16 ± 0.5	149.42 ± 30.5	164.27 ± 38.8	110.35 ± 46.5	12.53 ± 1.4	115.84 ± 34.0	15.35 ± 0.5
‘Senabija’	22.87 ± 4	120.50 ± 3.4	191.31 ± 10.1	161.22 ± 17.3	13.55 ± 1.7	106.04 ± 1.7	17.48 ± 1.2
‘Princeza’	30.77 ± 1.4	149.55 ± 7.0	203.40 ± 11.0	455.86 ± 2.1	4.98 ± 0.1	69.75 ± 1.4	15.46 ± 0.0
‘Kokos Reneta’	145.19 ± 8.5	141.21 ± 9.2	292.88 ± 18.9	247.68 ± 46.1	3.84 ± 0.7	52.74 ± 6.3	15.57 ± 1.3
‘Karlovčica’	33.72 ± 2.5	113.17 ± 16.1	169.57 ± 34.5	131.55 ± 42.1	0.70 ± 0.6	12.00 ± 3.8	3.54 ± 0.7
‘Zmazanka’	29.62 ± 2.8	191.27 ± 2.6	252.94 ± 3.7	165.20 ± 4.4	0.26 ± 0.2	18.35 ± 0.1	4.31 ± 0.1
‘Imperica’	31.50 ± 1.9	173.29 ± 15.1	228.39 ± 11.3	117.73 ± 10.5	1.43 ± 1.0	82.44 ± 3.5	13.91 ± 0.2
‘Šumatovka’	46.71 ± 3.6	161.90 ± 18.3	287.32 ± 16.9	204.29 ± 20.6	14.75 ± 1.6	93.89 ± 8.5	14.68 ± 1.2
‘Poglavnikova’	48.76 ± 4.5	161.43 ± 14.4	253.57 ± 3.9	169.04 ± 0.4	3.76 ± 0.9	47.40 ± 2.5	9.63 ± 0.2
‘Boskopska tikvica’	311.79 ± 18.1	216.50 ± 0.5	313.03 ± 1.5	244.43 ± 12.0	8.84 ± 1.0	132.19 ± 8.2	16.98 ± 0.8
‘Kablarka’	21.52 ± 29.9	135.25 ± 24.7	191.04 ± 29.1	297.40 ± 82.8	8.35 ± 4.0	25.50 ± 0.9	8.19 ± 0.2
‘Palaska’	95.29 ± 14.7	231.27 ± 41.8	284.83 ± 77.9	260.86 ± 91.3	15.69 ± 1.6	74.97 ± 12.5	8.81 ± 0.8
‘Bobovec’	132.15 ± 42.2	163.50 ± 46.2	285.99 ± 70.1	277.64 ± 166.0	11.43 ± 3.3	69.24 ± 30.1	16.95 ± 4.2
‘Bobovec Palči’	53.07 ± 6.3	169.91 ± 29.2	209.66 ± 26.8	281.06 ± 63.3	9.45 ± 2.8	87.89 ± 8.2	13.19 ± 1.1
‘Slastica’	40.12 ± 0.2	243.16 ± 28.5	230.50 ± 1.4	148.43 ± 10.8	5.11 ± 1.1	55.19 ± 4.1	9.58 ± 0.1
‘Gospoinjača’	0.70 ± 0.2	1.75 ± 0.1	4.08 ± 0.2	1.29 ± 0.2	0.15 ± 0.1	0.96 ± 0.1	0.70 ± 0.0
‘Kraljevača’	10.20 ± 13.9	127.31 ± 1.9	104.63 ± 1.1	328.57 ± 3.4	4.13 ± 1.6	36.55 ± 1.1	28.79 ± 0.3
‘Grofova’	10.87 ± 14.8	141.65 ± 5.2	167.73 ± 20.0	188.02 ± 19.5	0.82 ± 0.6	40.64 ± 0.4	12.90 ± 1.0
‘Meglena’	70.65 ± 46.3	119.68 ± 5.0	181.64 ± 28.5	245.67 ± 40.6	2.23 ± 1.4	21.78 ± 4.9	11.63 ± 1.1
‘Ilinjača’	120.65 ± 4.7	146.94 ± 15.8	256.56 ± 6.7	110.35 ± 46.5	4.39 ± 0.6	47.07 ± 2.7	15.53 ± 0.2
‘Ploska Letovanička’	67.72 ± 9.5	84.50 ± 8.8	148.96 ± 30.2	161.22 ± 17.3	2.41 ± 0.9	19.10 ± 8.1	11.49 ± 3.3
‘Ananas Reneta’	28.13 ± 2.8	86.13 ± 5.7	357.71 ± 35.2	455.86 ± 2.1	8.43 ± 1.0	230.66 ± 15.8	21.79 ± 1.1

^a^: Dry weight. Mean ± SD based on two extracts each measured twice (*n* = 4).

**Table 3:** Antioxidant activity (mmol TE/kg dw) of traditional apple varieties.

Sample ID	mmol TE/kg dw
‘Kolačarka’	23.9 ± 0.5
‘Muškatna Mirisava’	11.3 ± 0.4
‘Senabija’	12.1 ± 0.5
‘Princeza’	23.1 ± 1.3
‘Kokos Reneta’	27.0 ± 2.4
‘Karlovčica’	13.3 ± 2.3
‘Zmazanka’	14.7 ± 1.2
‘Imperica’	12,0 ± 1.1
‘Šumatovka’	36,2 ± 1.9
‘Poglavnikova’	29.7 ± 1.3
‘Boskopska tikvica’	35.6 ± 1.7
‘Kablarka’	29.3 ± 2.0
‘Palaska’	17.3 ± 1.3
‘Bobovec’	51.3 ± 3.7
‘Bobovec Palči’	17.7 ± 2.0
‘Slastica’	15.2 ± 1.0
‘Gospoinjača’	19.6 ± 1.7
‘Kraljevača’	16.1 ± 1.4
‘Grofova’	17.6 ± 0.9
‘Meglena’	59.0 ± 3.6
‘Ilinjača’	44.9 ± 4.2
‘Ploska Letovanička’	58.6 ± 3.4
‘Ananas Reneta’	35.6 ± 2.7

Mean ± SD based on two extracts, each measured twice (*n* = 4).

**Table 4:** Target polyphenols: CAS number, molecular mass, retention time and SRM transitions (precursor ion→ product ion (collision energy, eV)).

Polyphenols	CAS	Molecular Mass (g/mol)	Retention Time (min)	MS/MS Transitions ^1^
Gallic acid	149-91-7	170.1	2.61	169.02 → 125.04 (17) 169.02 → 153.1 (15)
2,4,6-trihydroxybenzoic acid	71989-93-0	188.1	4.22	168.98 → 150.99 (17) 168.98 → 83.02 (23) 168.98 → 107.02 (22)
2,4-dihydroxybenzoic acid	89-86-1	154.1	5.00	153.00 → 109.05 (16) 153.00 → 65.09 (19) 153.00 → 67.07 (23)
3,4-dihydroxybenzoic acid	99-50-3	154.1	5.00	152.98 → 109.04 (17) 152.98 → 91.04 (28) 152.98 → 108.03 (26)
Caftaric acid	67879-58-7	312.2	4.78	310.96→ 178.97 (17) 310.96 → 148.96 (14)
3,4-dihydroxybenzaldehyde	139-85-5	138.1	5.05	137.07 → 136.11 (21) 137.07 → 91.09 (24) 137.07 → 92.13 (25)
Procyanidin B1	20315-25-7	578.5	5.07	577.03 → 407.06 (26) 577.03 → 288.93 (25) 577.03 → 424.97 (26)
p-hydroxybenzoic acid	99-96-7	138.1	5.35	137.00 → 93.00 (17) 137.00 → 65.00 (27)
2,5-dihydroxybenzoic acid	490-79-9	117.1	5.38	152.96 → 108.00 (24) 152.96 → 81.02 (21) 152.96 → 109.01 (16)
Catechin	18829-70-4	290.3	5.50	289.00 → 245.02 (17) 289.00 → 203.11 (22)
3-hydroxyphenylacetic acid	621-37-4	152.2	5.70	151.00 → 65.00 (20) 151.00 → 79.00 (20)
Procyanidin B2	29106-49-8	578.5	5.96	577.03 → 407.06 (26) 577.03 → 288.93 (25) 577.03 → 424.97 (26)
2,5-dihydroxybenzaldehyde	1194-98-5	138.1	6.11	136.99 → 108.02 (21) 136.99 → 81.08 (18) 136.99 → 109.04 (14)
Chlorogenic acid	327-97-9	354.3	6.12	353.00 → 191.07 (22) 353.00 → 85.09 (43) 353.00 → 93.07 (45)
3-hydroxybenzaldehyde	100-83-4	122.1	6.22	121.02 → 93.05 (20) 121.02 → 92.05 (23) 121.02 → 120.04 (19)
4-hydroxybenzaldehyde	123-08-0	122.1	6.25	122.97 → 95.05 (13) 122.97 → 51.10 (36) 122.97 → 77.05 (20)
Vanillic acid	121-34-6	168.2	6.29	167.00 → 108.00 (27) 167.00 → 152.00 (18)
2,6-dihydroxybenzoic acid	303-07-1	154.1	6.33	153.00 → 109.05 (17) 153.00 → 65.09 (21) 153.00 → 135.02 (16)
3,5-dihydroxybenzoic acid	99-10-5	154.1	6.33	152.97 → 109.01 (15) 152.97 → 65.06 (16) 152.97 → 67.05 (20)
3,4-dimethoxybenzoic acid	93-07-2	182.2	6.45	182.96 → 137.08 (6) 182.96 → 106.99 (22)
Caffeic acid	331-39-5	180.2	6.50	178.98 → 135.03 (19) 178.98 → 134.01 (28)
Epicatechin	35323-91-2	290.3	6.56	289.00 → 245.02 (17) 289.00 → 203.11 (22)
Epigallocatechin gallate	989-51-5	458.4	6.79	457.15 → 169.05 (21) 457.15 → 125.09 (42) 457.15 → 305.09 (21)
Gallocatechin gallate	84650-60-2	458.4	7.29	457.15 → 169.05 (21) 457.15 → 125.09 (42) 457.15 → 305.09 (21)
Procyanidin A2	41743-41-3	576.5	7.32	577.09 → 287.00 (32) 577.09 → 136.98 (62) 577.09 → 425.08 (13)
7-hydroxycoumarin	93-35-6	162.1	7.80	162.99 → 107.04 (22) 162.99 → 77.05 (34) 162.99 → 91.05 (20)
p-coumaric acid	501-98-4	164.2	7.89	163.02 → 119.07 (18) 163.02→ 93.07 (37) 163.02 → 117.05 (38)
Catechin gallate	130405-40-2	442.3	8.01	441.13 → 289.13 (20) 441.13 → 125.08 (42) 441.13 → 169.05 (24)
Trans-ferulic acid	537-98-4	194.2	8.33	192.80 → 177.90 (12) 192.80 → 133.90 (16)
3,4-dimethoxybenzaldehyde	120-14-9	166.2	8.92	167.01 → 139.05 (13) 167.01 → 108.05 (21) 167.01 → 124.03 (18)
4-methoxybenzaldehyde	123-11-5	136.1	10.03	136.97 → 109.05 (12) 136.97 → 77.05 (23) 136.97 → 94.04 (18)
Quercetin-3-glucuronide	22688-79-5	478.4	10.32	479.09 → 461.50 (14) 479.09 → 302.96 (18)
Quercetin-3-rutinoside	153-18-4	610.5	10.35	609.18 → 270.92 (96) 609.18 → 178.87 (44) 609.18 → 300.01 (37)
Quercetin-3-glucoside	482-35-9	463.4	10.43	465.07 → 256.90 (41) 465.07 → 302.97 (14)
Myricetin	529-44-2	318.2	11.43	319.00 → 153.02 (31) 319.00 → 217.06 (31) 319.00 → 245.06 (27)
3,4,5-trimethoxycinnamic acid	90-50-6	238.2	11.59	239.03 → 221.04 (11) 239.03 → 162.99 (27) 239.03 → 190.01 (19)
3,5-dimethoxybenzaldehyde	7311-34-4	166.2	11.81	167.15 → 124.03 (17) 167.15 → 77.05 (26)
Quercetine	117-39-5	302.2	12.10	303.09 → 229.10 (28) 303.09 → 153.04 (33)
Kaempferol	520-18-3	286.2	12.57	285.07 → 184.91 (30) 285.07 → 239.12 (35)
Apigenin	520-36-5	270.2	12.63	269.09 → 117.12 (37) 269.09 → 149.12 (26) 269.09 → 151.06 (26)
Chrysin	480-40-0	254.2	13.24	253.13 → 143.18 (30) 253.13 → 63.20 (34) 253.13 → 145.16 (31)

^1^ Underlined MS/MS transition is used as quantification transition.

## Data Availability

The data presented in this study are available on request from the corresponding author.
